# Prevalence of parasitic contamination of raw vegetables in Nakhon Si Thammarat province, southern Thailand

**DOI:** 10.1186/s12889-018-6358-9

**Published:** 2019-01-08

**Authors:** Chuchard Punsawad, Nonthapan Phasuk, Kanjana Thongtup, Surasak Nagavirochana, Parnpen Viriyavejakul

**Affiliations:** 1 School of Medicine, Walailak University, 222 Thasala district, Nakhon Si Thammarat, 80161 Thailand; 2 Department of Tropical Pathology, Faculty of Tropical Medicine, Mahidol University, Bangkok, 10400 Thailand; 3 Tropical Medicine Research Unit, Research Institute for Health Sciences, Walailak University, 222 Thasala district, Nakhon Si Thammarat, 80161 Thailand

**Keywords:** Parasitic contamination, Raw vegetables, Intestinal parasites, Thailand

## Abstract

**Background:**

Soil-transmitted helminth (STH) infections are major public health problems in poor and developing countries that require fecal contamination of the environment for transmission. The consumption of raw vegetables without proper washing is one of the main routes of intestinal parasite acquisition. Therefore, this study was designed to detect the prevalence of intestinal parasitic contamination in commonly consumed raw vegetables sold in three central open-air markets in Nakhon Si Thammarat province, southern Thailand.

**Methods:**

A total of 265 fresh vegetable samples consisting of peppermint, lettuce, coriander, leek, gotu kola, celery, Chinese cabbage, culantro, Thai basil, and Chinese morning glory were purchased from three central open-air markets in the Mueang, Thasala and Sichon districts from December 2016 to March 2017. Each sample was washed with physiological saline, shaken for 15 min, and then allowed to sediment. Finally, sedimentation was performed via the sedimentation concentration technique and examined using light microscopy for the detection of pathogenic parasites.

**Results:**

The overall prevalence of parasitic contamination was 35.1% (93/265). The most predominant parasite was hookworms (42.9%), followed by *Strongyloides stercoralis* (10.6%)*, Trichuris trichiura* (2.6%), *Ascaris lumbricoides* (2.6%), and *Toxocara* spp. (2.6%). The highest level of contamination was found in celery, with a prevalence rate of 63.3% (19/30), while the lowest contamination level was found in Chinese morning glory, with a prevalence rate of 2.0% (2/30). The prevalence of intestinal parasite contamination in Mueang district (51.5%) was significantly higher than that in Thasala district (17.9%) and Sichon district (30.6%) (*P* < 0.001).

**Conclusion:**

The results of the present study demonstrate that consumption of vegetables with parasite contamination in this area represents a potential route for the transmission of parasitic infection, particularly hookworm infection. Therefore, it is necessary for health authorities to educate consumers about the proper washing of vegetables prior to consumption. Preventive methods such as wearing gloves and washing hands after handling vegetables should also be advocated to sellers who are at risk of acquiring STH infections via skin penetration.

## Background

Parasitic infections represent a major global public health problem. The burden of parasitic infections often affects developing countries, which frequently lack good sanitization and personal hygiene practices. Moreover, environmental factors such as climate, geography, temperature, soil type, and rainfall also play important roles that contribute to the prevalence of parasitic infections.

In southern Thailand, the most prevalent parasitic infection among all age groups has been soil-transmitted helminth (STH) infections, especially hookworm infection [1–4], which may transmit to humans through either skin penetration or the ingestion of its larvae [5].

The consumption of fresh vegetables is a common eating habit among people in southern Thailand. Further, healthy food trends in Thailand are now focused on sufficient daily vegetable consumption, as this could help prevent major diseases, such as cardiovascular disease and certain cancers. The WHO also recommended the intake of a minimum of 400 g of fruit and vegetables per day for micronutrient supplementation as well as the prevention of the aforementioned chronic diseases [6]. However, eating raw vegetables may also lead to the transmission of certain human pathogens (fresh fruits and vegetables are vectors for the transmission of human pathogens) [7]. Several studies have reported the parasitic contamination of fresh vegetables in many countries around the world, for example, Arba Minch town, southern Ethiopia [8], Khartoum state, Sudan [9], Benha, Egypt [10], Accra, Ghana [4], Mazandaran province, northern Iran [11], Poland [12], and Metro Manila, Philippines [13]. Parasites and protozoa that were common contaminants included *Ascaris lumbricoides*, *Cryptosporidium* spp., *Entamoeba histolytica*/*dispar*, *Enterobius vermicularis*, *Giardia intestinalis*, hookworm, *Hymenolepis* spp., and *Trichuris trichiura* [4, 8–12, 14]. These contaminants could result from water used to moisten vegetables and postharvesting handling methods [8, 9, 15].

Nakhon Si Thammarat province is an endemic area for STH infection [3]. To date, there have been no reports on parasitic vegetable contamination in this area. Therefore, the main aim of this study was to determine the prevalence of parasitic contamination in commonly consumed vegetables in Nakhon Si Thammarat province, southern Thailand.

## Methods

### Study area

**Fig. 1:**
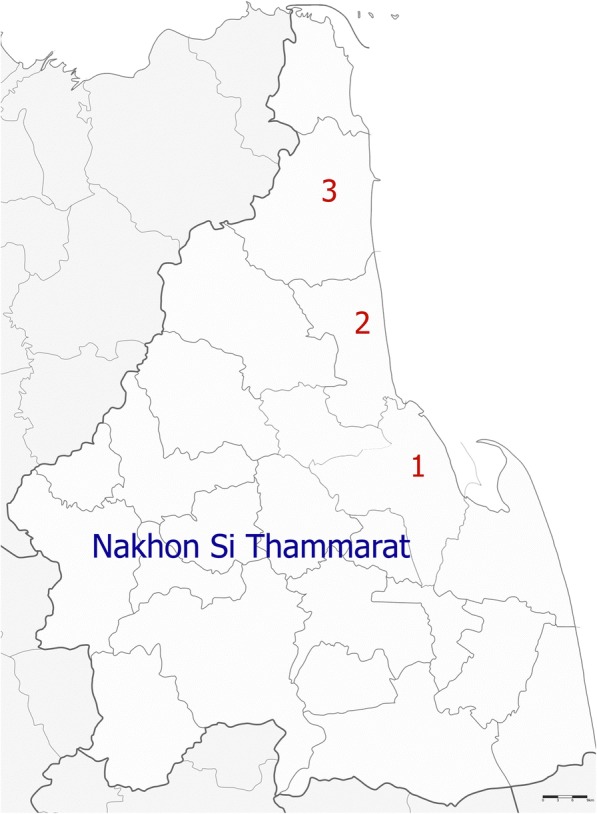
Location of the study area including the Mueang (1), Thasala (2) and Sichon districts (3) of Nakhon Si Thammarat province, southern Thailand (Map from Wikimedia Commons: https://commons.wikimedia.org/wiki/File:Amphoe_8001.svg)

A cross-sectional study was conducted from December 2016 to March 2017 in Nakhon Si Thammarat province, which is located in southern Thailand approximately 800 km from Bangkok, the capital of Thailand. This area has a tropical rainforest climate and is located at the geographical coordinates of 8°43′10″N latitude and 99°45′6″E longitude. The average annual temperature is 28.2 °C, and approximately 1702.6 mm of precipitation falls annually (Climatological Center, Thai Meteorological Department, Annual report 2015). According to official statistic registration systems, the total population in this area is approximately 1,557,482. Fresh vegetable samples were purchased from three central open-air markets located in Mueang (the capital district of Nakhon Si Thammarat province), Thasala (approximately 30 km away from the capital district) and Sichon districts (approximately 70 km away from the capital district) (Fig. 1). The fresh vegetables sold in these markets were brought from different farms and agricultural areas in different parts of Nakhon Si Thammarat province.

### Sample collection

**Fig. 2:**
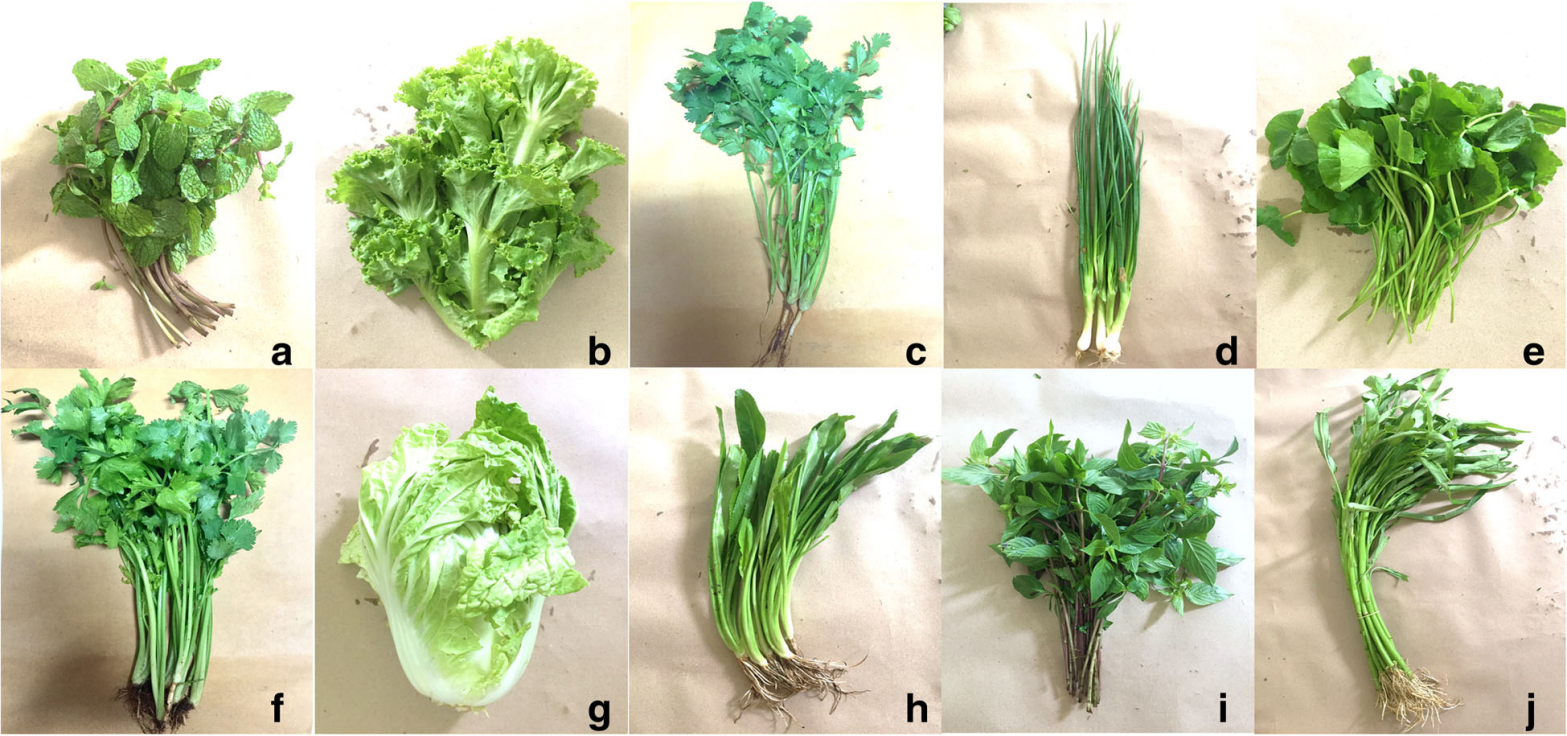
The pictures of vegetable samples including **a**. peppermint (*Mentha x piperita*), **b**. lettuce (*Lactuca sativa*), **c**. coriander (*Coriandrum sativum*), **d**. leek (*Allium porrum*), **e**. gotu kola (*Centella asiatica*), **f**. celery (*Apium graveolens*), **g**. Chinese cabbage (*Brassica rapa subsp. pekinensis*), **h**. culantro (*Eryngium foetidum*), **i**. Thai basil (*Ocimum basilicum*), and **j**. Chinese morning glory (*Ipomoea aquatica*)

A total of 265 fresh vegetable samples including 10 different types that are frequently consumed without cooking were randomly purchased from sellers in three central open-air markets. The fresh raw vegetable samples used in this study included peppermint (*Mentha x piperita*), lettuce (*Lactuca sativa*), coriander (*Coriandrum sativum*), leek (*Allium porrum*), gotu kola (*Centella asiatica*), celery (*Apium graveolens*), Chinese cabbage (*Brassica rapa subsp. pekinensis*), culantro (*Eryngium foetidum*), Thai basil (*Ocimum basilicum*), and Chinese morning glory (*Ipomoea aquatica*). The pictures of vegetable samples were demonstrated in Fig. 2. The fresh vegetable samples were collected in clean, labeled plastic bags and transported immediately to the parasitology laboratory at the School of Medicine, Walailak University for parasitic examination.

### Detection of intestinal parasites

Fresh vegetable samples weighing 200 g were washed with 1000 mL of physiological saline solution (0.9% sodium chloride) and shaken for 15 min in order to separate the parasites from vegetables. Then, the washing water was collected and left overnight to allow sedimentation. Afterward, the supernatant was decanted, and the remaining washing water was transferred to 12 mL conical tubes. To concentrate the parasitic stages, the sediment was centrifuged at 2000×g for 15 min. After centrifugation, the supernatant was carefully removed without shaking. Then, the sediment was agitated gently and examined under a light microscope using 10× and 40× objectives. To increase the chance of parasite detection, three slides were prepared from each sample by two independent investigators.

### Statistical analysis

Data analysis was performed with IBM SPSS Statistics for Windows, Version 23.0. Armonk, NY: IBM Corp. Qualitative variables were described by frequency (percentage). A chi-squared test was used to compare the rate of parasitic contamination among different types of vegetable and among different markets. A *p*-value less than 0.05 was considered statistically significant.

## Results

**Fig. 3:**
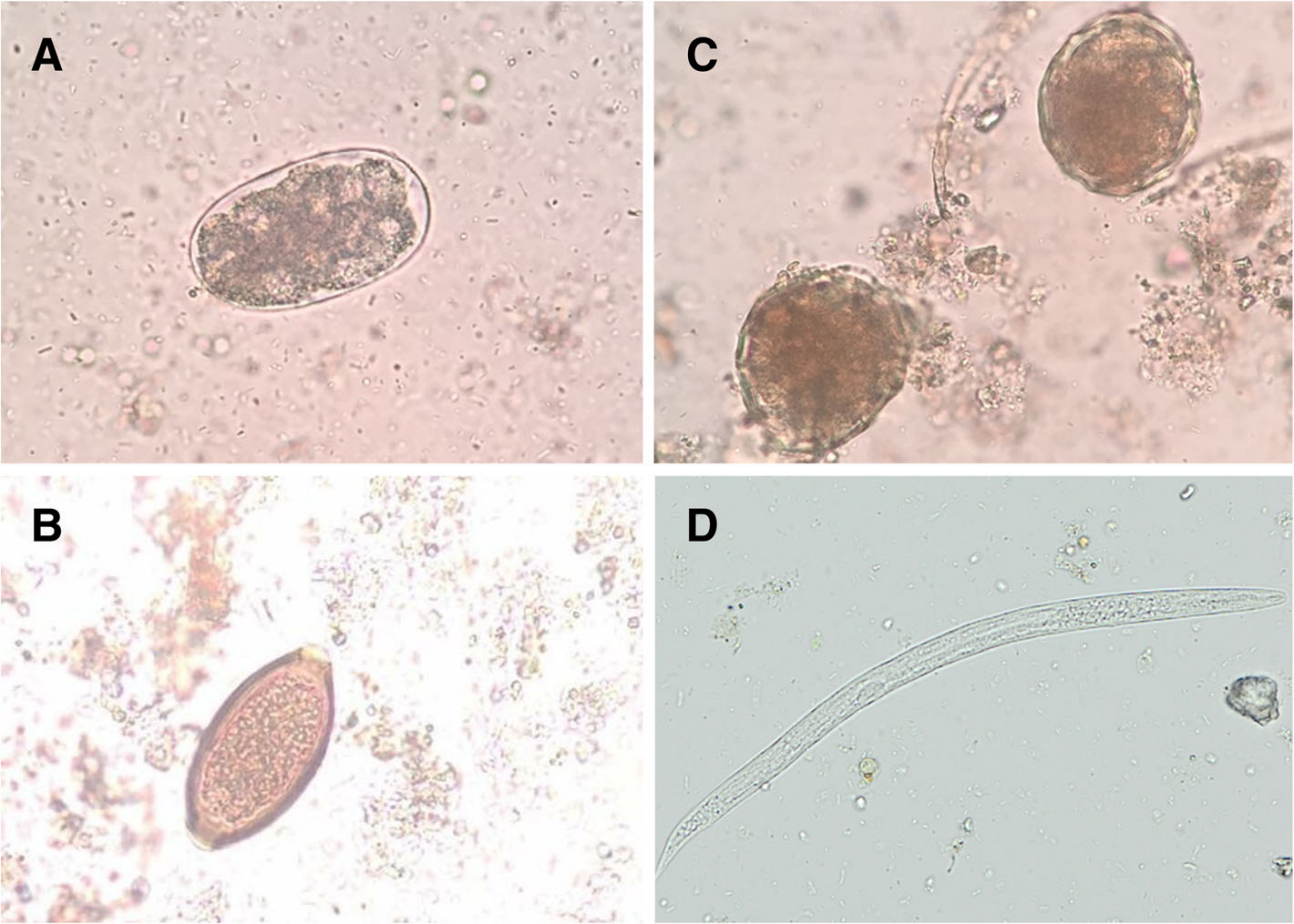
Representative images of parasites found in this survey. Hookworm egg (**a**), *Trichuris trichiura* egg (**b**), *Ascaris lumbricoides* eggs (**c**) and *Strongyloides stercoralis* larvae (**d**). Original magnification: 40X

A total of 265 fresh vegetable samples were examined for the presence of parasite contamination. The results of parasitic contamination in vegetable samples are shown in Table 1. The parasites detected in vegetable samples were hookworm, *Strongyloides stercoralis*, *Trichuris trichiura*, *Ascaris lumbricoides*, and *Blastocystis* spp. Pictures of some of the parasites found in this survey were demonstrated in Fig. 3. The overall rate of intestinal parasite detection was 35.1% (93/265) in all vegetable samples. The highest rate of contamination was found in celery [63.3% (19/30)] while the lowest was found in Chinese morning glory [6.8% (2/30)] (Table 1). The highest rate of hookworm contamination was found in celery, but not Thai basil or Chinese morning glory. Strongyloides larvae were most frequently detected in celery but in lettuce. Eggs of *Trichuris trichiura* were frequently detected in culantro but were not found in peppermint, lettuce, coriander, leek, gotu kola, or celery. Eggs of *Ascaris lumbricoides* were frequently detected in lettuce, coriander, and celery.

**Table 1:** Distribution of intestinal parasites in fresh vegetable samples collected from three markets in Nakhon Si Thammarat province, Thailand

Vegetables	Number of sample	Hookworm Eggs (%)	*Strongyloides stercoralis* larvae	*Trichuris trichiura* eggs	*Ascaris lumbricoides* eggs	*Toxocara* spp. eggs	Number positive (%)^a^
Peppermint	15	6 (40.0)	2 (13.3)	0	0	1 (6.7)	9 (60.0)
Lettuce	20	1 (5.0)	0	0	3 (15)	0	4 (20.0)
Coriander	29	8 (27.6)	2 (6.9)	0	3 (10.3)	0	13 (44.8)
Leek	30	7 (23.3)	6 (20.0)	0	0	0	13 (43.3)
Gotu kola	21	7 (33.3)	4 (19.0)	0	0	1 (4.8)	12 (57.1)
Celery	30	9 (30.0)	9 (30.0)	0	1 (3.3)	0	19 (63.3)
Chinese cabbage	30	2 (6.7)	1 (3.3)	2 (6.7)	0	2 (6.7)	7 (23.3)
Culantro	30	4 (13.3)	2 (6.7)	3 (10.0)	0	2 (6.7)	11 (36.7)
Thai Basil	30	0	1 (3.3)	1 (3.3)	0	1 (3.3)	3 (10.0)
Chinese morning glory	30	0	1 (3.3)	1 (3.3)	0	0	2 (6.7)
Total	265	44 (16.6)	28 (10.6)	7 (2.6)	7 (2.6)	7 (2.6)	93 (35.1)

^a^ Number of positive parasitic contamination events are significantly different among types of vegetables (*P* < 0.001)

Table 2 shows the prevalence of parasitic contamination in different vegetable samples among the three markets. The parasites were detected in 51.5%, 30.6%, and 17.9% of samples obtained from Mueang, Thasala, and Sichon districts, respectively. The highest rate of parasitic contamination in vegetables was found in Mueang district, whereas the lowest rate was observed in Thasala district. Statistical analysis revealed that the rate of contamination in vegetables obtained from Mueang district was significantly higher than those obtained from Sichon and Thasala district (*P* < 0.001).

**Table 2:** Distribution of intestinal parasite contamination in different fresh vegetables among the three markets in Nakhon Si Thammarat province, Thailand

Vegetable type	No. of examined samples		Mueang district		Thasala district		Sichon district	
	Examined	Positive (%)	No. of examined	No. of positive (%)	No. of examined	No. of positive (%)	No. of examined	No. of positive (%)
Peppermint	15	9 (60.0)	11	7 (63.6)	0	0 (0.0)	4	4 (40.0)
Lettuce	20	4 (20.0)	10	4 (40.0)	6	0 (0.0)	4	0 (0.0)
Coriander	29	13 (44.8)	10	8 (80.0)	9	1 (11.1)	10	4 (40.0)
Leek	30	13 (43.3)	10	6 (60.0)	10	4 (40.0)	10	3 (30.0)
Gotu kola	21	12 (57.1)	10	7 (70.0)	3	2 (66.7)	8	3 (37.5)
Celery	30	19 (63.3)	10	9 (80.0)	10	4 (40.0)	10	6 (60.0)
Chinese cabbage	30	7 (23.3)	10	3 (30.0)	10	1 (10.0)	10	3 (30.0)
Culantro	30	11 (36.7)	10	5 (50.0)	10	1 (10.0)	10	5 (50.0)
Thai Basil	30	3 (10.0)	10	2 (20.0)	10	0 (0.0)	10	2 (20.0)
Chinese morning glory	30	2 (6.7)	10	1 (10.0)	10	1 (10.0)	10	0 (0.0)
Total	265	93 (35.1)	101	52 (51.5)^a^	78	14 (17.9)^a^	85	26 (30.6)^a^

^a^ Number of positive parasitic contamination events are significantly different among three markets (*P* < 0.001)

## Discussion

The consumption of raw vegetables plays an important role in the transmission of parasites to humans. The recovery of parasites from vegetables helped us better understand the potential source of pathogenic parasite acquisition in this study area. This study demonstrated that the prevalence of parasitic contamination in vegetable samples from Nakhon Si Thammarat province was 35.1%, which was comparable to the prevalence of 32.6% and 34.7% from previous reports in Shahrekord, Iran [16] and Poland [17], respectively. However, higher contamination rates, ranging from 45% to 58%, were detected in Libya, Iran, Egypt, Brazil, and the Philippines [8, 13, 18–22]. In contrast, the rate of contamination was lower in some studies, such as 5.9% in Ankara, Turkey [23], 14.89% in Mazandaran province, northern Iran [11], 19.4% in Alexandria, Egypt [24], and 26% in Hanoi, Vietnam [25]. According to the influence of season on the prevalence of parasites, previous studies reported that the rate of STH contamination was higher in warm seasons versus cold seasons [11, 19, 26]. This finding suggested that the transmission and prevalence rate of parasites were associated with climate and temperature. Differences between this study and others might be attributed to variations in climatic conditions, types of soil, types of water used for agriculture, and poor hygienic practices during the transportation and marketing of vegetables.

Celery was found to be the most frequently contaminated sample (63.3%) followed by peppermint (60.0%), gotu kola (57.1%), coriander (44.8%), leek (43.3%), culantro (36.7%), Chinese cabbage (23.3%), lettuce (20%), and Thai basil (20%), whereas Chinese morning glory (6.7%) was the least contaminated. These results were different from previous studies, which reported that lettuce possessed the highest parasitic contamination [9, 10, 21]. In Thailand, celery is commonly used for seasoning and garnishing in many Thai dishes. The highest contamination in celery might be because celery consists of a number of stalks that are connected at the base with its leaves near the top of stalks. The structure of its stalk is roughly U-shaped in a slit pattern, which allows the parasites to attach more easily to the surface of these vegetables and makes them more difficult to remove. Normally, celery samples obtained from the three markets were frequently sold with roots and stalks, which might increase the chance of soil contamination from roots to stalks. In contrast, the lowest parasitic contamination was observed in Chinese morning glory, which could be because the smooth surface of its stalks that may reduce the probability of parasitic attachment.

In this present study, hookworm was detected in 16.60% (44/265) of vegetables examined and was the most predominant pathogenic parasite. This finding is in agreement with other studies conducted in Ghana (13%) [4], Sudan (5.7%) [9], and northern Iran (4.40%) [11]. In contrast, no ova of hookworm species were recovered in some previous studies [8, 10, 16, 18–20, 22–25, 27]. Our previous report in 2017 demonstrated that hookworm infection was the most prevalent parasitic infection in Nopphitam district, Nakhon Si Thammarat province, southern Thailand [2]. The high prevalence of hookworm egg contamination of vegetables in this study could be attributed to poor sanitation and the use of human waste-contaminated water for irrigation in the region. Lack of proper footwear and exposure of skin to contaminated soil might be responsible for hookworm infection in the study area. In addition, the high prevalence of hookworm might be due to differences in geographical location, climate conditions, and the types of soil [28, 29]. The second most prevalent contamination found in this study was the larvae of *Strongyloides stercoralis*. Our finding was consistent with previous studies in Accra, Ghana and in Jimma Town, Ethiopia, which reported a 43% and 21.9% prevalence of *Strongyloides stercoralis* contamination, respectively [4, 22]. A high rate of *Strongyloides* contamination might be because *Strongyloides* spp. has a complex life cycle with a free-living stage in the environment that does not require a host for its proliferation [30].

Aside from the contamination of hookworm eggs, *T. trichiura* and *Ascaris lumbricoides* eggs were also detected in the vegetable samples collected from this area. Trichuriasis occurs by ingestion of contaminated food and water with embryonated eggs of *T. trichiura* [31]. In the present study, eggs of *T. trichiura* were detected in 2.64% (7/265) of vegetable samples. This finding was consistent with previous reports from Khartoum state, Sudan [9], Mazandaran province, northern Iran [11], Accra, Ghana [4] and in villages of Qazvin province, Iran [27], where the contamination rates were 2.9, 2.2, 2 and 0.9%, respectively. In addition, eggs of *Ascaris lumbricoides* were detected in 2.64% (7/265) of vegetable samples. The rate of contamination with *A. lumbricoides* eggs was 68% in Tripoli, Libya [18], 20.83% in Arba Minch town, southern Ethiopia [8] and 8.17% in Shahrekord, Iran [26]. Contamination by these STHs in vegetable samples might occur at any point along the chain; during planting, harvesting, transportation, or the marketing of vegetables. The differences in the degree of contamination might be attributed to the types of soil, the quality of water used for planting and irrigation, and hygienic practices during the process of marketing.

Beside STH contamination, this study also detected *Toxocara* spp. eggs in 2.64% (7/265) of fresh vegetable samples. In other locales, the rate of contamination with *Toxocara* spp. eggs in vegetables was 37% in Libya [18], 15.83% in Arba Minch town, southern Ethiopia [8], 3.3% in Shahrekord, Iran [16], 3% in Hanoi, Vietnam [25], and 1.5% in Ankara, Turkey [23]. In contrast, no ova of *Toxocara* spp. in vegetables were reported in Ardabil, Khorramabad, and Qasvin, Iran, Accra, Ghana, Benha and Alexandria, Egypt, Minas Gerais, Brazil, or Khartoum state, Sudan [4, 9, 10, 19–21, 24, 27]. Human toxocariasis is a helminthic zoonosis caused by larval stages of *Toxocara canis* and less frequently by *Toxocara cati* [32]. The long term survival of *Toxocara* spp. outside their hosts coupled with their high fecundity is responsible for significant contamination of soil with infective eggs [32]. This study indicates that domestic animals (dogs and cats), which are the source of *Toxocara* eggs, may at some point shed contaminated feces onto cultivation areas.

The parasitic contamination rates were significantly different for samples collected from different markets; samples collected from Muang district showed the highest rate of contamination. The differences between the three markets might be due to the different sources of vegetables as well as the hygienic practices in handling and washing by different sellers.

The results of this study emphasized that raw vegetables from the markets in the study area could be possible vehicles of parasitic transmission to humans. The previous studies revealed that the standard washing procedure was an effective method to prevent the contaminations of helminths in raw vegetables [16, 23, 33]. Furthermore, to emphasize on the proper washing procedure, the previous study in Iran demonstrated that the pre-washing procedure using tap water or underground water could not completely get rid of parasites from vegetables [26]. Hence, the health authorities should provide the knowledge of proper washing method for local people in order to prevent parasitic transmission.

However, it is important to note that our study has several limitations. This study did not demonstrate the effect of seasonal variation on parasitic contamination. We did not address the intensity of vegetable washing before display for sale or the source of water used by each seller. These factors might affect the rate of vegetable contamination in our study.

## Conclusion

The results of the present study indicate that the contamination of raw vegetables with pathogenic parasites in Nakhon Si Thammarat province, southern Thailand might represent a transmission vector for intestinal parasites to consumers. Prevention methods such as proper washing or cooking of vegetables before consumption should be conveyed to consumers. In addition, comprehensive health education and hygienic practices, including wearing gloves and washing hands after handling vegetables, should be provided to sellers and farmers.

## Abbreviations

STH: Soil-transmitted helminth
WHO: World Health Organization
